# New Insights on End-Stage Renal Disease and Healthy Individual Gut Bacterial Translocation: Different Carbon Composition of Lipopolysaccharides and Different Impact on Monocyte Inflammatory Response

**DOI:** 10.3389/fimmu.2021.658404

**Published:** 2021-06-07

**Authors:** Hanane Adda-Rezig, Clémence Carron, Jean-Paul Pais de Barros, Hélène Choubley, Émilie Charron, Anne-Laure Rérole, Caroline Laheurte, Pascale Louvat, Émilie Gaiffe, Dominique Simula-Faivre, Valérie Deckert, Laurent Lagrost, Philippe Saas, Didier Ducloux, Jamal Bamoulid

**Affiliations:** ^1^ Univ. Bourgogne Franche-Comté, INSERM, EFS BFC, UMR1098, RIGHT Interactions Hôte Greffon-Tumeur/Ingénierie Cellulaire et Génique, Fédération Hospitalo-Universitaire INCREASE, LabEx LipSTIC, Besançon, France; ^2^ INSERM, Univ. Bourgogne Franche-Comté, LNC UMR1231, LabEx LipSTIC, Dijon, France; ^3^ EFS Bourgogne Franche-Comté, Plateforme de BioMonitoring, Besançon, France; ^4^ INSERM CIC1431, University Hospital of Besançon, Clinical Investigation Center in Biotherapy, Fédération Hospitalo-Universitaire INCREASE, LabEx LipSTIC, Besançon, France; ^5^ University Hospital of Besançon, Department of Nephrology, Dialysis, and Renal Transplantation, Besançon, France

**Keywords:** end-stage renal disease, gut bacterial translocation, lipid A (LPS), HDL - cholesterol, chronic inflammation

## Abstract

Chronic kidney disease induces disruption of the intestinal epithelial barrier, leading to gut bacterial translocation. Here, we appreciated bacterial translocation by analyzing circulating lipopolysaccharides (LPS) using two methods, one measuring only active free LPS, and the other quantifying total LPS as well as LPS lipid A carbon chain length. This was done in end-stage renal disease (ESRD) patients and healthy volunteers (HV). We observed both higher LPS concentration in healthy volunteers and significant differences in composition of translocated LPS based on lipid A carbon chain length. Lower LPS activity to mass ratio and higher concentration of high-density lipoproteins were found in HV, suggesting a better plasma capacity to neutralize LPS activity. Higher serum concentrations of soluble CD14 and pro-inflammatory cytokines in ESRD patients confirmed this hypothesis. To further explore whether chronic inflammation in ESRD patients could be more related to LPS composition rather than its quantity, we tested the effect of HV and patient sera on cytokine secretion in monocyte cultures. Sera with predominance of 14-carbon chain lipid A-LPS induced higher secretion of pro-inflammatory cytokines than those with predominance of 18-carbon chain lipid A-LPS. TLR4 or LPS antagonists decreased LPS-induced cytokine production by monocytes, demonstrating an LPS-specific effect. Thereby, septic inflammation observed in ESRD patients may be not related to higher bacterial translocation, but to reduced LPS neutralization capacity and differences in translocated LPS subtypes.

## Introduction

End-stage renal disease (ESRD) is associated with persistent elevated plasma concentrations of pro-inflammatory cytokines. Patients with ESRD have a higher risk to acquire infections than the general population, and septicemia is one of the most severe type of infection (1). The progressive accumulation of uremic toxins is supposed to contribute to loss of the intestinal epithelial barrier integrity, gut bacterial translocation (GBT), dysbiosis, and chronic inflammation (2, 3). Exposure to bacterial structures, such as lipopolysaccharides (LPS) yields an inflammatory response mediated by innate immunity (4). This inflammatory response to LPS in ESRD has been demonstrated to be potentialized by uremic toxins and contributes to altered immune response dysfunctions observed in chronic kidney disease (CKD) (5).

LPS is a major structural component of the outer membrane of Gram-negative bacterial cell wall. It is composed of three structural domains: an amphipathic lipid A, a core oligosaccharide and an O-antigen polysaccharide (6). The lipid A structure is conserved at the species level and plays a major role in bacterial pathogenicity (7) and immunogenicity (8). LPS is considered as a main pathogen-associated molecular pattern (PAMP) (9).

Cellular response to LPS is mediated by interactions between LPS-binding protein (LBP), CD14, and myeloid differentiation factor-2 (MD-2), which lead to the dimerization of the Toll-like receptor-4 (TLR4) (10). LBP is required to extract LPS monomers from bacterial outer membrane or from LPS aggregates (11). Then, LPS monomers are delivered to MD-2/TLR4 for its activation and dimerization of the LPS/MD-2/TLR4 ternary complex (12), leading to the production of pro-inflammatory cytokines (13). This transfer of LPS to MD-2/TLR4 requires the concerted action of extracellular LBP with soluble (s) or membrane-bound (m) CD14 provides (14). Dimerization of the LPS/MD-2/TLR4 ternary complex is less efficient depending on LPS structural variants, such as LPS with Phe^126^ in fatty acyl chains (15). Complexes with such LPS variants require higher concentrations to induce a similar TLR4-dependent cell activation (15). In fact, the optimal recognition of LPS by the MD-2/TLR4 complex occurs when the lipid A moiety of LPS has 6 fatty acyl chains and 2 phosphates. This structure corresponds to LPS produced by most *Enterobacteriaceae* (8). Moreover, some lipid A moieties are not able to induce an inflammatory signal, but rather inhibit the host response to more immunogenic LPS varieties. Therefore, LPS structures, and in particular lipid A structures, influence the ability to trigger host immune responses (16, 17).

Circulating LPS clearance is mainly mediated by high-density lipoprotein (HDL) (18). LBP facilitates the binding of LPS to HDL for its transfer to the liver, and therefore its inactivation (19). Thus, binding to HDL inhibits the ability of LPS to interact with MD2/TLR4 complex and prevents monocyte/macrophage activation (20). In addition to neutralizing circulating LPS, HDL can also facilitate the release of LPS already bound to myeloid cells, reducing their activation (21). However, in the setting of low circulating HDL, LBP allows LPS transfer to CD14 resulting in immune cell activation (20). Thus, HDL plays a key role in LPS neutralization.

Nowadays, little is known on the LPS structure and the impact of the different circulating LPS in human cells. The *Limulus amebocyte* lysate (LAL) assay is currently the most frequent method for detection of circulating free LPS (22). However, this LAL assay better reflects LPS bioactivity than the complete LPS “picture” (8, 23). Until now, effective blood measurements of total LPS are scarce in clinical studies. Quantitative total LPS analysis is now possible using mass spectrometry (MS/MS) coupled to high performance liquid chromatography (HPLC) based on lipid A 3-hydroxymyristate (3-HM) quantification (24). This method measures total circulating LPS, including bound and unbound LPS to lipoproteins and not only LPS activity (*i.e.*, unbound LPS able to activate the LAL assay). This has been used in different pathological settings (25, 26). Yet, the quantification of LPS subtypes according to the length of lipid A carbon chains (3OHC12:0, 3OHC14:0, 3OHC16:0 or 3OHC18:0) has never been tested in a pathogenic setting.

We previously demonstrated that the concentration of 3-HM (corresponding to 3OHC14:0 LPS, *i.e.*, 14-carbon chain lipid A-LPS) was higher in ESRD patients and remained stable one year after kidney transplantation (KT). Yet, LPS activity and inflammation biomarkers decreased 1-Year post KT whereas we concomitantly observed an increase in circulating lipoproteins (27). In this study, we aimed to revisit LPS metabolism analysis in ESRD patients in comparison to healthy volunteers (HV). We analyzed circulating LPS subtypes (3OHC12:0, 3OHC14:0, 3OHC16:0, 3OHC18:0), as well as their influence on the inflammatory response. Firstly, we measured both LPS circulating mass and LPS bioactivity. Secondly, we explored the capacity of both populations to neutralize LPS. Finally, we used an *in vitro* model of monocyte culture to test whether the distribution of LPS subtypes present in ESRD or HV sera could influence the inflammatory response and whether the TLR4 pathway was implicated in the observed inflammatory process.

## Materials And Methods

### Study Design and Plasma Samples

The GABII (*Globulines Anti-lymphocytaires polyclonales et Barrière Immunitaire Intestinale*) cohort (NCT02843841, protocol number P/2014/221) is a prospective, longitudinal study designed to assess the effect of anti-thymocyte globulins on gut barrier and microbiota in kidney transplantation setting. Exclusion criteria were an infectious episode within the last month before transplantation, an ongoing antibiotherapy at time of inclusion, or a chronic infectious disease (hepatitis B or C viruses, or HIV). Sixty-eight consecutive patients have been enrolled (**Table 1**). Samples were collected and sent to the Biomonitoring Platform (*Etablissement Français du Sang* (EFS), Besançon, France) for processing and storage. For each patient, blood samples were collected prior to transplantation (ESRD) after at least 6 hours of fast, either from fistula when hemodialysis was required before transplantation (n=17 among 43 patients on hemodialysis) or from peripheral vein (n=51, *i.e.*, 26 on hemodialysis, 16 on peritoneal dialysis and 9 preemptive kidney transplant candidates). Blood was also collected several times after transplantation (four days, three months and one year after at least 6 hours of fast). Plasma samples were isolated by centrifugation and cryopreserved. For the present study, only ESRD samples were analyzed. The sample collection was approved by the French Ministry of Higher Education and Research (agreement number #DC-2008-713). The study was approved by the local Ethics Committee (CPP-EST-II, Besançon, France): favorable opinion ethic 14/438 of May 13^th^, 2014 and regulatory authorization n°140578B-11 of July 18^th^, 2014 (ANSM reference: 2014-A00424-43). Plasma samples were also collected from twenty healthy anonymous volunteers at the EFS (Besançon, France) (**Table 1**) after at least 6 hours of fast. No participants were under antibiotherapy at time of inclusion, and all were free from a chronic infectious disease (see above). Patients enrolled in the GABII study and healthy volunteers (HV) gave their written informed consent.

**Table 1:** Characteristics of end-stage renal disease (ESRD) patients from GABII cohort and healthy volunteers (HV) included in this study.

GABII cohort	ESRD patients				*p*	HV
	Total	Hemodialysis	Peritoneal dialysis	Preemptive patients		
**N**	68	43	16	9	_	20
**Age (years)**	51±12	54±14	45±15	53±15	0.115	43±10
**Male gender (%)**	42 (62)	24 (56)	12 (75)	6 (67)	0.394	18 (90)
**BMI (kg/m²)**	26.3±4.2	26.3±5.3	27.7±6.7	24.3±2.2	0.325	25.8±2.3
**Dialysis duration (months)**	33±26	37±27	23±19	_	0.056	_
**Previous kidney transplantation n. (%)**	9 (13)	7 (16)	1 (6.3)	1 (11)	0.600	_
**Diabetes n. (%)**	16 (24)	13 (30)	2 (13)	1 (11)	0.239	_
**Hypertension n. (%)**	67 (98.5)	43 (100)	15 (93)	9 (100)	0.184	_
**Dyslipidemia n. (%)**	36 (53)	21 (49)	9 (56)	6 (67)	0.606	_
**Current smokers n. (%)**	15 (22)	14 (33)	0 (0)	1 (11)	0.022	_
**Medication with statins n. (%)**	28 (41.2)	16 (37.2)	7 (43.8)	5 (55.6)	0.630	_
**Medication with platelet antiaggregant n. (%)**	10 (14.7)	8 (18.6)	2 (12.5)	0 (0)	0.355	
**Medication with phosphate-binding agent n. (%)**	42 (61.8)	29 (67.4)	11 (68.8)	2 (22)	0.015	
**Anti-HLA immunization n. (%)**	17 (25)	12 (28)	4 (25)	1 (11)	0.583	_
**Etiology of kidney disease n. (%)**					0.288	
*Chronic glomerulopathy*	14 (21)	8 (19)	5 (31)	1 (11)		_
*Nephroangiosclerosis*	4 (6)	2 (5)	0 (0)	2 (22)		_
*Polykystic disease*	9 (13.5)	5 (12.5)	3 (18)	1 (11)		_
*Diabetes*	5 (7.5)	4 (9.5)	1 (6)	0 (0)		_
*Other hereditary nephropathy*	1 (1.5)	0 (0)	1 (6)	0 (0)		_
*Other nephropathy*	22 (32)	14 (35)	6 (36)	2 (22)		_
*Unknown*	13 (19)	10 (25)	0 (0)	3 (33)		_
**CKD-EPI Estimated GFR (ml/min/1.73m2)**	8.7±4.0	8.4±3.8	7.2±3.2	13.5±3.6	0.002	_
**Dialysis session before transplantation**	33 (48.5)	17 (39.5)	16 (100)	0 (0)	0.0001	_

Results are presented as mean ± SD. BMI, body mass index; CKD-EPI, chronic kidney disease - epidemiology collaboration; GABII, Globulines Anti-lymphocytaires polyclonales et Barrière Immunitaire Intestinale; GFR, glomerular filtration rate; HLA, human leukocyte antigen; M/F ratio, male/female ratio.

### Endotoxemia

Biological activity of LPS was quantified in plasma samples by the end-point chromogenic *Limulus amebocyte* lysate (LAL) assay (Hycult Biotech, Uden, Netherlands). Quantification of plasma total LPS was performed using LPS-derived or 3HM –a fatty acid of lipid A– extracted from plasma samples with an organic solvent, separated and quantitated by simultaneous HPLC/MS/MS analysis (24). In human samples, LPS-derived/esterified 3HM was calculated as the difference between total 3HM and unesterified/free 3HM. Here, four LPS subtypes were determined depending on lipid A carbon chain length (3OHC12:0, 3OHC14:0, 3OHC16:0, 3OHC18:0, thereafter named: C12, C14, C16, C18, respectively), their sum was considered as total LPS.

### Soluble Factors, Lipoproteins and Cytokines

Soluble CD14 was measured in plasma samples using Quantikine enzyme-linked immunosorbent assay (ELISA) kit (R&D Systems, Minneapolis, MN) according to the manufacturer’s recommendations. Plasma lipoproteins were assayed using commercially available kits (Cholesterol and HDL-cholesterol, Thermo Fisher Scientific, Finland) on an Indiko Clinical Chemistry analyzer (Thermo Fisher Scientific, Finland) according to the manufacturer’s instructions. Phospholipid transfer protein (PLTP) activity was measured in serum using a commercially available fluorescence activity assay from Roar Biomedical (New York, NY, USA), according to the manufacturer’s instructions. The concentrations of tumor necrosis factor α (TNF-α), interleukin (IL)-1β, IL-6 and IL-10 were determined in plasma samples by using a Human Magnetic Luminex assay (R&D Systems, Minneapolis, MN USA) according to the manufacturer’s instructions. Standards and samples were analyzed on a LuminexR® apparatus (Bio-Plex 200, BioRad, München, Germany) using the BioPlex Manager Software (Version 5, BioRad, Hercules, CA).

### Stimulation and Culture of Monocytes

Blood samples were collected from healthy anonymous volunteers at the *Etablissement Français du Sang* (EFS, Besançon, France) with written informed consent. Peripheral blood mononuclear cells (PBMCs) and sera were isolated by density gradient centrifugation using Ficoll-Paque^TM^. Autologous serum was decomplemented by heating at 56°C for 30 minutes. The isolation of CD14^+^ monocytes was assessed by magnetic separation using a commercially kit (CD14 MicroBeads human, MACS Miltenyi Biotech, GmbH) according to manufacturer’s recommendations. Monocytes were cultured in RPMI media supplemented with penicillin/streptomycin (100 μg/mL each) and 7% of decomplemented autologous serum (AS), or ESRD sera according to the highest proportion of each LPS subtypes (P/C12, P/C14, P/C16, P/C18, n=3 for each), or HV sera (n=5), or without serum (WS, negative control, n=3). For the positive control, LPS from *E. coli* (LPS-EK, 10 ng/mL, InvivoGen, USA) was added to the complete media supplemented with AS. To demonstrate the involvement of the TLR4 pathway, LPS-EK or sera (P/C12, P/C14, P/C16, P/C18, HV) were co-cultured with a TLR4 antagonist (LPS from *R. sphaeroides*, LPS-RS, 100 ng/mL, InvivoGen, USA) or an antibiotic blocking TLR4 signaling (Polymyxin B, PMB, 100 µg/mL, InvivoGen, USA). The different conditions were performed in duplicate. After 12 hours of stimulation, supernatants were collected and the concentration of cytokines (TNF-α, IL-1β, IL-6 and IL-10) were measured by ELISA kits (Biolegend, USA), according to manufacturer’s recommendations. We ascertained cytokine bias measurement, due to basal concentrations of cytokines that were potentially present in tested sera, by systematic subtraction of basal concentrations of each cytokine –previously measured in sera before addition to the monocyte cultures– from the cytokine concentration measured in collected supernatants at the end of monocyte cultures. Thus, the cytokine concentrations reported in the manuscript were all ‘net’ concentrations.

### Statistical Analysis

All results are depicted as median with interquartile range (median [interquartile]) or mean with standard deviation (mean ± SD). The Mann-Whitney U test was used for pairwise comparison. The correlation studies were assessed with non-linear regression (Spearman) and represented with linear regression. Differences with a *p-value* below 0.05 were considered statistically significant (*p<0.05; **p<0.01; ***p<0.001; ****p<0.0001). Data were performed using GraphPad Prism 5 (GraphPad Software, San Diego, CA).

## Results

### Characteristics of ESRD Patients and Healthy Volunteers

Demographics and clinical characteristics are summarized in **Table 1**. Nine patients were not dialyzed (preemptive kidney transplant candidates) with a mean serum creatinine of 470±207 µmol/L (mean creatinine clearance according to CKD-EPI formula of 12.22±4.35ml/min/1.73m^2^), 43 were on hemodialysis and 16 were on peritoneal dialysis. Seventeen patients out of 43 under hemodialysis were dialyzed before transplantation. Comparisons of patient’s characteristics according to dialysis modality did not show any significant differences between the 3 groups.

### LPS Is Detectable in ESRD and in Healthy Setting, but LPS Subtype Composition Differs Between ESRD Patients and HV

**Figure 1:**
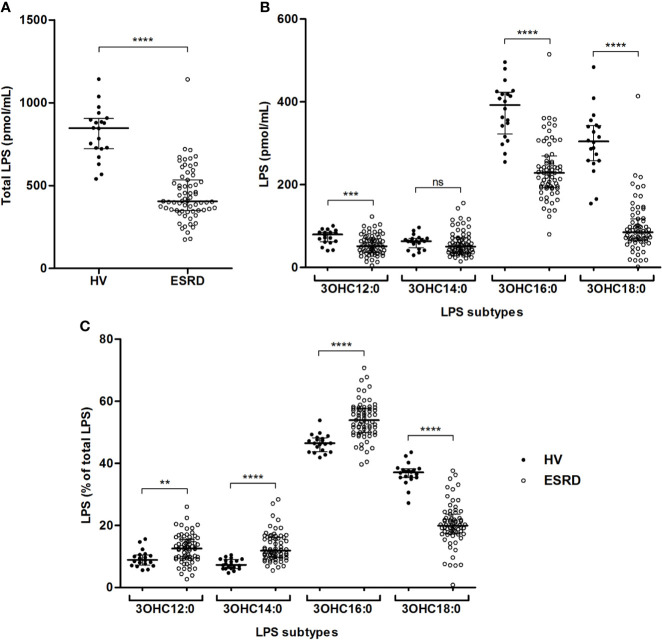
Gut bacterial translocation in end-stage renal disease (ESRD) patients and healthy volunteers (HV). Comparison of total circulating LPS **(A)**, concentrations **(B)** and proportions **(C)** of circulating LPS subtypes between ESRD patients (n = 68) and healthy volunteers (n = 20). LPS quantities were measured in serum by HPLC/MS/MS method. LPS subtypes differed according to the length of lipid A carbon chains (3OHC12:0, 3OHC14:0, 3OHC16:0, 3OHC18:0) and their sum was considered as the total circulating LPS concentration used to determine LPS proportions. Bars and errors bars are expressed as median with interquartile range. Mann-Whitney U test of HV *versus* ESRD patient’s values, **p < 0.01, ***p < 0.001, ****p < 0.0001, ns, not significant. HPLC/MS/MS, high performance liquid chromatography (HPLC) coupled with mass spectrometry (MS/MS); LPS, lipopolysaccharide.

We simultaneously quantified four circulating LPS subtypes according to the length of the carbon chain composing the lipid A present in ESRD patients and HV. The total LPS concentration, corresponding to the sum of these four LPS subtypes, was higher in HV (846.7 [723.0–905.1] *vs* 404.9 [349.6–534.4] pmol/mL; *p*<0.0001) (**Figure 1A**). LPS concentrations were higher in HV samples for three out of the four LPS subtypes (**Figure 1B**). This difference was observed for C12, C16 and C18 LPS, but not for C14 LPS. Comparing mean percentages of LPS subtypes between ESRD patients and HV (**Figure 1C** and **Supplementary Table S1**), sera of ESRD patients exhibited higher mean proportion of C12, C14 and C16 LPS, while HV sera exhibited higher proportion of C18 LPS.

### LPS Clearance Is Lower in ESRD Than in Healthy Volunteers

**Figure 2:**
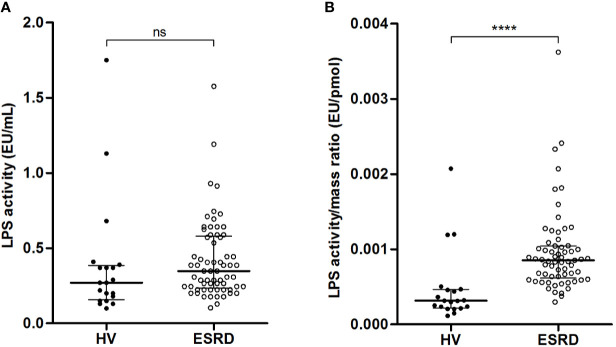
Relationship between quantity and activity of circulating LPS in end-stage renal disease (ESRD) patients and healthy volunteers (HV). Comparison of LPS activity **(A)** and LPS activity to mass ratio **(B)** between ESRD patients (n = 68) and healthy volunteers (n = 20). LPS quantity and LPS activity were measured in serum by HPLC/MS/MS method and LAL test respectively. Bars and errors bars are expressed as median with interquartile range. Mann-Whitney U test of HV *versus* ESRD patient’s values, ****p < 0.0001, ns, not significant. HPLC/MS/MS, high performance liquid chromatography (HPLC) coupled with mass spectrometry (MS/MS); LAL, *Limulus amebocyte* lysate, LPS, lipopolysaccharide.

LPS activity was measured using the LAL test (**Figure 2A**). There was no significant difference between HV and ESRD patients (0.27 [0.16–0.39] *vs.* 0.35 [0.23–0.58] EU/mL; *p*=0.0789). Noteworthy, the LPS activity to mass ratio (**Figure 2B**) was significantly lower in HV than ESRD patients (3.1x10^E-4^ [2.2x10^E-4^–4.7x10^E-4^] *vs.* 8.5x10^E-4^ [6.2x10^E-4^–1.0x10^E-3^] EU/pmol; *p*<0.0001). Thus, HV exhibit more circulating bound/neutralized LPS than ESRD patients.

Lipids, and particularly HDL, play a major role in LPS clearance. Lipid analyses were performed in both groups. There was no difference in total cholesterol (1.85 [1.76–2.05] *vs.* 1.80 [1.45–2.11] g/L; *p*=0.3204). Yet, the concentrations of HDL-cholesterol were significantly higher in HV, as compared with ESRD patients (0.55 [0.43–0.65] *vs.* 0.42 [0.31–0.51] g/L; *p*=0.0007). Conversely, the plasma PLTP activity was higher in ESRD patients (258.1 [198.7–335.0] *vs.* 170.3 [149.0–195.0] pmol/h; *p*<0.0001). Thus, more HDL lipoproteins are available in HV than ESRD patients to neutralize circulating LPS.

### LPS-Induced Inflammation Is Greater in ESRD Patients

**Figure 3:**
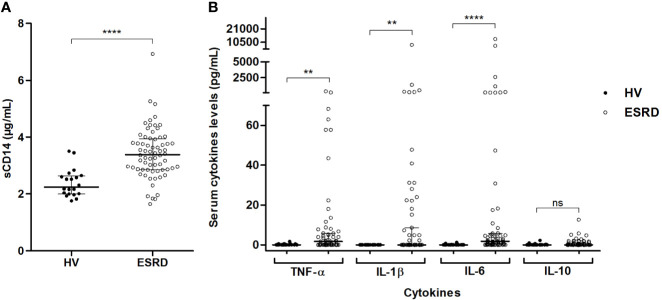
LPS-induced inflammation in end-stage renal disease (ESRD) patients and healthy volunteers (HV). Comparison of sCD14 **(A)** and inflammatory cytokines **(B)** serum levels between ESRD patients (n = 68) and healthy volunteers (n = 20). Soluble CD14 and cytokines (TNF-α, IL-1β, IL-6, IL-10) were determined in serum by enzyme-linked immunosorbent assay (ELISA) and Luminex methods respectively. Bars and errors bars are expressed as median with interquartile range. Mann-Whitney U test of HV *versus* ESRD patient’s values, **p < 0.01, ****p < 0.0001, ns, not significant. IL, interleukin; LPS, lipopolysaccharide; sCD14, soluble cluster of differentiation 14; TLR4, Toll-like receptor-4; TNF, tumor necrosis factor.

To confirm that free (bioactive) LPS circulate in a higher concentration in ESRD patients, soluble form of CD14 (sCD14) –that reflects host immune cell LPS-induced activation– and pro-inflammatory cytokines were measured. In ESRD samples, sCD14 (**Figure 3A**), as well as TNF-α, IL-1β and IL-6 (**Figure 3B**) were significantly increased (**Supplementary Table S2**). This confirms the increase of active LPS and suggests an LPS-induced inflammation in ESRD patients.

### Proportion of C18 LPS in Total LPS Is Inversely Correlated With LPS-Induced Inflammation

**Figure 4:**
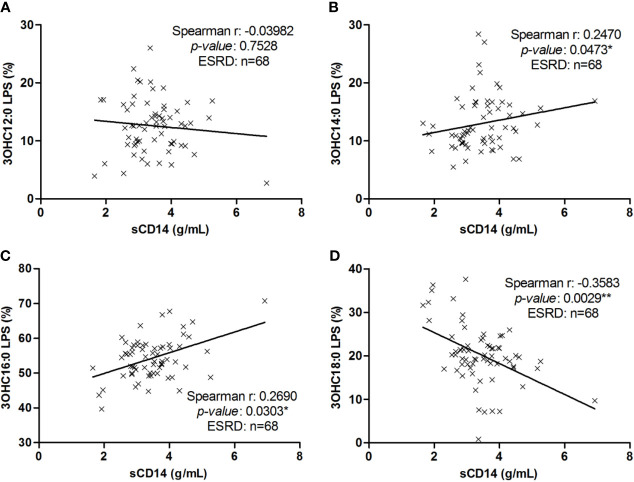
Correlation between LPS subtype’s proportion with sCD14 in end-stage renal disease (ESRD) patients. Correlation between sCD14 serum levels and 3OHC12:0 **(A)**, 3OHC14:0 **(B)**, 3OHC16:0 **(C)** or 3OHC18:0 **(D)** LPS proportions in ESRD patients (n = 68). Each cross represents a patient. Linear regression was displayed on graphs. Spearman correlation between sCD14 and LPS proportions, *p < 0.05, **p < 0.01. LPS, lipopolysaccharide; sCD14, soluble cluster of differentiation 14.

We explored the correlation between sCD14 concentrations (a reflect of LPS-induced TLR4 stimulation) and each LPS subtype proportions in ESRD samples (**Figure 4**). Soluble CD14 was positively related to percentage of C14 LPS (r=0.2470; *p*=0.0473) (**Figure 4B**) and inversely to percentage of C18 LPS (r=-0.3583; *p*=0.0029) (**Figure 4D**). However, there was no significant correlation between sCD14 concentrations and C12 (r=-0.0398; *p*=0.7528) or C16 (r=0.2351; *p*=0.0615) LPS proportions (**Figures 4A, B** **)**.

The analysis of C14 and C18 LPS percentage revealed a strong negative correlation (r=-0.699; *p*<0.0001) between both LPS subtypes. The percentage of C18 LPS was inversely correlated with concentrations of IL-7 (r=-0.47; *p*<0.0001), proportion of C14 (r=-0.47; *p*<0.0001) and C16 LPS (r=-0.45; *p*<0.0001). The concentration of C18 LPS was inversely correlated with concentrations of sCD14 (r=-0.29; *p*=0.02), TNF-α (r=-0.31; *p*=0.01), IL-7 (r=-0.37; *p*=0.002), C14 (r=-0.45; *p*<0.0001) and C16 LPS (r=-0.79; *p*<0.0001), and positively correlated with concentration of C12 LPS (r=0.53; *p*<0.0001).

We defined 2 clusters among ESRD patients according to C18/(C12+C14+C16) LPS ratio. Cluster 1 (n=51) was defined with a center value of 22% and cluster 2 (n=17) with a center value of 42% (*p*<0.001). Clinical characteristics and treatments of cluster 1 were comparable to those of cluster 2 ESRD patients (**Supplementary Table S3**). Comparison of cluster 1 to cluster 2 ESRD patients showed significant differences regarding sCD14 (3.51 [3.02-3.96] *vs.* 2.86 [1.93-3.50] pg/mL; *p*=0.002), TNF-α (1.80 [0.0-8.0] *vs* 0.00 [0.00-2.51] pg/mL; *p*=0.028), IL-8 (119.4 [3.40-664.03] *vs.* 26.56 [9.30-264.21] pg/mL; *p*=0.027) and IL-7 (2.74 [1.20-5.56] *vs.* 1.64 [1.33-2.02] pg/mL; *p*=0.002). This demonstrated a strong statistical relationship between C18/(C12+C14+C16) LPS ratio and LPS-induced inflammation.

Therefore, we hypothesized that C14 LPS has a pro-inflammatory effect, while C18 LPS has anti-inflammatory properties. These correlations were not found in the HV group (**Supplementary Figure S1**).

### Monocyte Stimulation With Sera Containing a High C18 LPS Proportion Was Less Prone to Secrete High Amount of TLR4-Induced Pro-Inflammatory Cytokines

**Figure 5:**
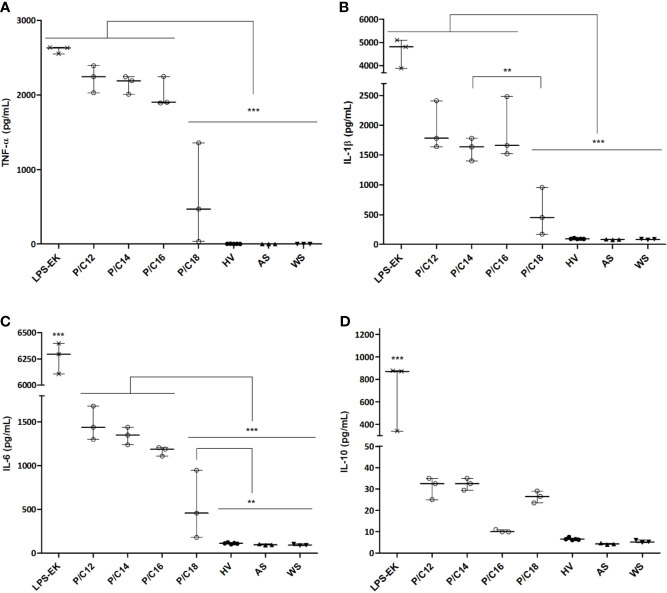
Cytokine concentrations in supernatants of monocytes cultured with sera of end-stage renal disease (ESRD) patients (n = 12) according to the highest proportion of each LPS subtype and healthy volunteers (HV) (n = 5). Human monocytes were stimulated with ESRD patient’s serum according to the highest proportion of each LPS subtypes (P/C12, P/C14, P/C16, P/C18, n = 3 for each), with serum of HV (n = 5), with decomplemented autologous serum (AS, n = 3), with AS and LPS from *E. coli* (LPS-EK, 10ng/mL, n = 3) for the positive control, or monocytes cultured without serum (WS, n = 3) for the negative control. After 12 hours of stimulation, supernatants were collected and the secretion of cytokines TNF-α **(A)**, IL-1β **(B)**, IL-6 **(C)** and IL-10 **(D)** were measured by enzyme-linked immunosorbent assay (ELISA). Bars and errors bars are expressed as median with interquartile range. Mann-Whitney U test of pairwise comparison values, **p < 0.01, ***p < 0.001. IL, interleukin; LPS, lipopolysaccharide; TNF, tumor necrosis factor.

To ascertain the influence of the major LPS subtype measured in ESRD samples on the GBT-associated inflammatory state, we stimulated *in vitro* monocytes with different sera. ESRD patient samples were selected according to the highest proportion of each LPS subtypes (C12, C14, C16, and C18, n=3 for each) (**Supplementary Tables S4, S5**). Sera from HV (n=5) (**Supplementary Table S6**), decomplemented sera autologous to monocytes (AS, n=3), autologous sera spiked with LPS from *E. coli* (LPS-EK, 10 ng/mL) were used as positive controls, while a negative control consisted in absence of serum. After 12 hours of stimulation, supernatants were collected and cytokines (TNF-α, IL-1β, IL-6 and IL-10) were measured (**Figure 5**). First, LPS-EK, a powerful TLR4 agonist, induced the highest cytokine secretion (TNF-α: 2630.0 [2552.0–2637.0] pg/mL; IL-1β: 4807.0 [3890.0–5102.0] pg/mL; IL-6: 6295.0 [6106.0–6396.0] pg/mL; and IL-10: 872.5 [339.7–878.6] pg/mL; *p*<0.001 compared to all other culture conditions for any cytokine). Concentrations of pro-inflammatory cytokines were significantly higher when stimulated with C14 LPS sera as compared with C18 LPS stimulation (TNF-α: 2190.0 [2008.0–2243.0] *vs.* 470.5 [38.0–1357.0] pg/mL, *p*=0.0008; IL-1β: 1636.0 [1401.0–1780.0] *vs.* 450.5 [171.0–956.0] pg/mL, *p*=0.0029; and IL-6: 1350.0 [1239.0–1439.0] *vs.* 458.5 [181.0–949.0] pg/mL, *p*=0.0009). Hence, monocytes stimulated with serum containing the highest proportion of C18 LPS were less prone to secrete high amounts of pro-inflammatory cytokines. The cytokine levels were close to those measured in monocytes stimulated with HV serum (TNF-α: 470.5 [38.0–1357.0] *vs.* 3.2 [2.6–4.2] pg/mL, *p*=0.0678; IL-1β: 450.5 [171.0–956.0] *vs.* 93.8 [90.0–100.5] pg/mL, *p*=0.1459; IL-6: 458.5 [181.0–949.0] *vs* 112.6 [105.3–120.1] pg/mL, p=0.009; and IL-10: 26.5 [23.5–29.0] *vs.* 6.5 [6.2–7.0] pg/mL, *p*=0.2109). Thus, sera containing a highest proportion of C18 LPS are less able to trigger pro-inflammatory cytokines than sera containing a highest proportion of C14 LPS.

**Figure 6:**
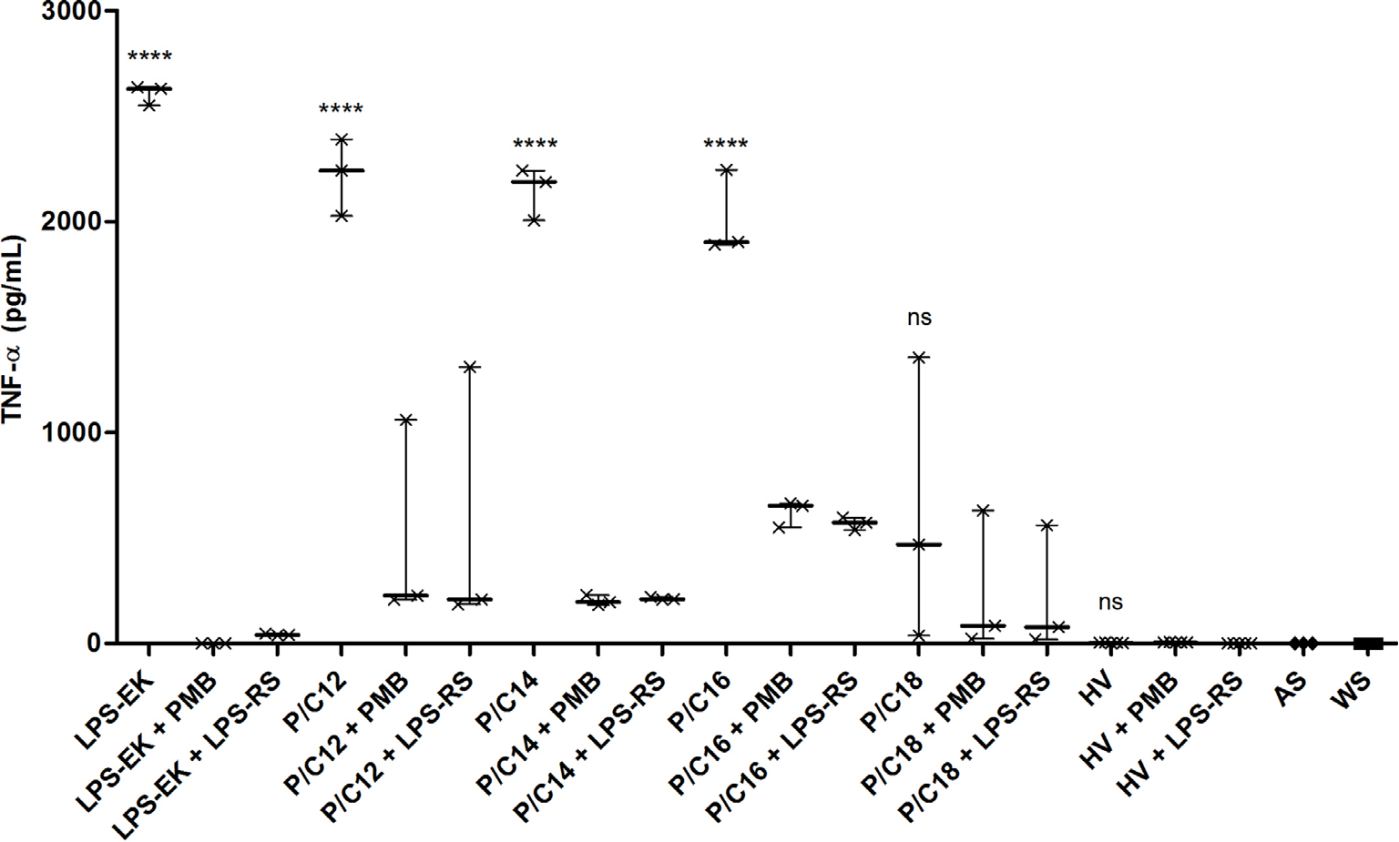
TLR4-dependent inflammation pathway in end-stage renal disease (ESRD) patients and healthy volunteers (HV). Human monocytes were stimulated with ESRD patient’s serum according to the highest proportion of each LPS subtypes (P/C12, P/C14, P/C16, P/C18, n = 3 for each), with HV serum (n = 5), or with decomplemented autologous serum and LPS from *E. coli* (LPS-EK, 10ng/mL, n = 3) for the positive control. Moreover, cells and sera were cultured with an antagonist of TLR4 (LPS from *R. sphaeroides*, LPS-RS, 100 ng/mL) or an antibiotic blocking LPS activity (Polymyxin B, PMB, 100 µg/mL). After 12 hours of stimulation, supernatants were collected and the secretion of cytokines TNF-α was measured by enzyme-linked immunosorbent assay (ELISA). Bars and errors bars are expressed as median with interquartile range. Mann-Whitney U test of single stimulation (LPS-EK or sera) and co-stimulation with PMB or LPS-RS values, ****p < 0.0001, ns, not significant; LPS, lipopolysaccharide; TLR4, Toll-like receptor-4; TNF, tumor necrosis factor.

To demonstrate the specific implication of the TLR4 pathway in cytokine production by monocytes, the different sera were treated with a TLR4 pathway antagonist (LPS from *Rhodobacter sphaeroides*, LPS-RS, 100 ng/mL) or a blocker of LPS activity through binding to lipid A (Polymyxin B, PMB, 100 µg/mL). After 12 hours of stimulation, the same cytokines (TNF-α, IL-1β, IL-6 and IL-10) were measured in the collected supernatants (**Figure 6** and **Supplementary Figure S2**). Addition of PMB or LPS-RS induced a significant decrease in the secretion of pro-inflammatory cytokines in all the conditions tested (*p*<0.0001), except in the case of sera enriched in C18 LPS. This demonstrates the direct involvement of the TLR4 pathway in the cytokine response of monocytes stimulated with different LPS subtypes containing serum.

## Discussion

In summary, this study demonstrates that GBT –assessed by circulating LPS– is not synonymous of pathology, since we detected lower total amounts of circulating LPS in ESRD than in healthy individuals. This suggests no significant increase in Gram-negative bacterial translocation in ESRD patients. By contrast, the capacity to neutralize circulating LPS and circulating LPS subtypes greatly differs between ESRD patients and HV. Our results suggest that septic inflammation observed in ESRD is not related to increased gut permeability and subsequent LPS translocation, but to both a shift towards more inflammatory LPS subtypes and reduced capacity to neutralize LPS.

Total circulating LPS concentration measured by HPLC/MS/MS was higher in HV. However, the composition of translocated LPS is different, with a predominance of circulating C18 LPS in HV. Although the total amount of circulating LPS was higher in HV, its activity –assessed by LAL assay– remained relatively lower. This attests a better LPS neutralization in HV. This was further explained by both higher concentrations of HDL in HV, and higher PLTP activity in ESRD patients. Higher circulating free LPS in ESRD may induce an increased PLTP activity, in order to transport free LPS to HDL However, low HDL concentration reduces the capacity of LPS neutralization in ESRD patients, as compared with HV. The lack of hepatic LPS clearance may favor LPS-induced inflammation in ESRD patients. Indeed, we observed an increase in LPS-induced inflammation biomarkers (as attested by sCD14, IL-1β, IL-6, TNF-α) and an increased TLR4-mediated cytokine release by monocytes cultured with ESRD serum in comparison to HV serum. Moreover, monocytes stimulated with serum containing the highest proportion of C18 LPS were less prone to secrete high amounts of pro-inflammatory cytokines. Cytokine levels found after stimulation with C18 LPS-enriched sera from ESRD patients were found similar than those measured after monocyte stimulation with HV serum. This confirms that composition and equilibrium of circulating LPS subtypes rather than total circulating LPS quantity directly influence LPS-induced inflammation. Thereby, the pathogenicity of circulating LPS depends more on its subtype composition and on the plasma neutralization capacities than on total LPS concentrations.

Biomarkers of gut bacterial integrity are difficult to interpret in the setting of ESRD. Although we also quantified plasma iFABP (intestinal fatty-acid binding protein) concentrations (**Supplementary Figure S3A**) –an intracellular protein specifically and abundantly expressed in the epithelial cells of the mucosal layer of the small and large intestine tissue (28) and a non-invasive biomarker for evaluating gut wall integrity loss– in both groups, we chose to not include these data to our report. iFABP is released into the circulation following intestinal mucosal disruption. Thus, its plasma concentrations have been associated with small intestinal disease such as coeliac disease (29). In this study, we observed a basal iFABP plasma level in healthy individuals, reflecting the physiological turnover rate of enterocytes. On the contrary, increased concentrations were observed in ESRD patients and might indicate intestinal epithelial cell disruption (30, 31) (0.2200 [0.1300–0.4500] *vs.* 2.121 [1.462–3.205] ng/mL; *p*<0.0001). To determine whether iFABP plasma levels could be used as a marker of the intestinal epithelial cell damage, we evaluated the correlation between plasma iFABP and GBT using the total circulating LPS quantification (**Supplementary Figure S3B**). Plasma iFABP concentrations were positively correlated with total circulating LPS (r=0.2895; *p*=0.0393). Thus, the gut barrier integrity seems to be disrupted, suggesting an increase in intestinal permeability in ESRD. Nevertheless, we also observed that iFABP concentrations were inversely correlated with glomerular filtration rate measured one-year post-transplant (r*=*-0.335; *p=*0.009), confirming our previous reported study (27). These concordant results suggest that plasma dosage of biomarkers of gut integrity could be misinterpreted in the setting of CKD and rather be the reflect of renal function than of gut integrity. Of note, other gut integrity biomarkers such as zonulin –an enterocyte tight junction protein– may also be influenced by renal function. Indeed, serum zonulin was correlated with the secretion of sucrose in urine in a recent report (32). Moreover, other reports demonstrated that zonulin was inversely correlated with creatinine (33) or was significantly lower among patients with chronic kidney disease in comparison with healthy volunteers (34). On the contrary, Ficek and co-workers reported that plasma levels of zonulin were significantly higher in ESRD than in healthy controls (35).

Several studies observed human microbiome dysregulation in CKD/ESRD, revealed by either quantitative and qualitative changes of the gut microbiota (36). The gut microbiome is constituted by more than 50 bacterial phyla, with *Bacteroidetes* and *Firmicutes* being the most prevalent (34). In health, the gut microbiome coexists with the host in a symbiotic manner, conferring trophic and protective functions. In disease, this equilibrium is altered and dysbiosis occurs leading to both qualitative and quantitative changes of intestinal microbiota. In general, the variations in CKD/ESRD context more often described in current studies were increases in *Enterobacteriaceae* (Gram-negative bacteria) and decreases in both *Bifidobacterium spp.* and *Lactobacillus spp.* (Gram-positive bacteria) (37). This microbiota imbalance may be due to intestinal availability of uremic toxins and other factors (metabolic acidosis, intestinal wall edema, slow colonic transit, pharmacological therapies or dietary restrictions) disturbing this symbiotic relationship and contributing *in fine* to uremic toxicity, inflammation, and cardiovascular risk in CDK/ESRD patients (38). In normal conditions, uremic toxins which bind with plasma albumin are excreted into urine, and unbound toxins are removed through glomerular filtration. However, in patients with CKD/ESRD such elimination of toxins is reduced due to the renal impairment which results in their blood accumulation (39). This accumulation was suggested to be related to enhanced generation of toxins from the dysbiotic microbiome accompanied by their reduced elimination by impaired kidneys. Intestinal microbiota plays a key role in the accumulation of uremic toxins (numerous uremic solutes are generated in the process of protein fermentation by colonic microbiota) and the presence of CKD may be accompanied by the development of intestinal inflammation and epithelial barrier impairment leading to hastened systemic translocation of bacterial-derived uremic toxins (39). In CKD, the impairment of the intestinal barrier function may enable the translocation of intestinal microorganisms (endotoxin, antigens, and other microbial products to the intestinal wall such as LPS), suggesting that ESRD patients commonly exhibit histological evidence of chronic inflammation throughout the gastrointestinal tract with endotoxemia and systemic inflammation (36).

In a previous work of our group, we reported higher concentrations of circulating 3-hydroxytetradecanoic acid (3-hydroxymyristate or 3-HM) –corresponding to C14 LPS–in ESRD patients (n=146) as compared with HV (n=11) (27). This result seems contradictory with what we reported in the present study. Yet, the previous results were obtained from the ORLY-Est cohort which is a multicentric study with samples collected from seven French Transplant Centers, while the GABII cohort was a monocentric study specifically designed to study GBT in ESRD patients. Although the samples collection was performed by the same biomonitoring platform in both studies, the patients fast could not be guaranteed in the ORLY-Est cohort and the conditions and time variation in samples processing and storage could have influenced the C14 LPS concentrations. Moreover, as we hypothesized a relationship between GBT and acute rejection (AR), we included more patients with AR from the ORLY-Est cohort while we included all consecutive included patients whatever their AR status in the GABII cohort. Finally, we reported here that C14 LPS subtype concentration was correlated with age (r=0.244; *p*=0.023). Of note, in the previous work, ESRD population was older than HV (50.0 *vs* 38.7 y-o; *p*=0.004) and only 11 HV were included (27), while, here, we better matched ESRD and HV for age (51.0 *vs.* 43.0; *p*=0.115) and increased the number of included HV (n=20) in the GABII cohort. Thus, the monocentric and consecutive inclusion (*vs* multicentric and selected according to AR status) design, the differences in sampling, transportation time and storage conditions and the use of numerous sera from older HV in the present study could explain the differences observed in C14 LPS concentrations between the two studies.

The HPLC/MS/MS method brings new complementary information on the quantity and subtypes of LPS, opening new insights on GBT exploration. The diversity of lipid A structures has a significant impact on its bioactivity, because it alters the recognition of LPS by the TLR4/MD2 complex (40). Some Gram-negative pathogens, such as *Helicobacter pylori*, *Legionella pneumophila* and *Chlamydia trachomatis*, synthesize lipid A molecules that are poorly recognized by TLR4 (41). For example, *Francisella tularensis* lipid A is composed of 18-carbon chains (C18) with poor stimulation capacity of TLR4 (42), whereas the lipid A of *Escherichia coli*, accounting for 12- or 14-carbon chains, is a powerful activator of TLR4 and therefore of the innate immune system (7). Vatanen and co-workers recently studied biological effects of LPS extracted from *Escherichia coli* and *Bacteroides dorei* on the production of pro-inflammatory cytokines (TNF-α, IL-1β, IL-6 and IL-8) (43). They demonstrated that LPS from *Bacteroides dorei* reduces pro-inflammatory cytokine production induced by LPS from *Escherichia coli*. Thus, LPS from *Bacteroides dorei* acts as an inhibitor of the immune stimulation by LPS derived from *Escherichia coli* (43), as LPS from *Rhodobacter sphaeroides* used in our study. Nowadays, the structure of lipid A has not been described yet for all bacteria; nevertheless, these findings clearly distinguish two types of LPS: those with immuno-stimulatory lipid A structures and those with inhibitory lipid A structures. This may influence the overall host immune response.

The endotoxic activity of LPS generally relies on the number, length and distribution of lipid chains along the disaccharide backbone of lipid A as well as on the phosphorylation status of the sugar units. A canonical endotoxic lipid A of *Escherichia coli* is hexa-acylated (the lipid chains entail 12 to 14 carbon atoms) and possesses two phosphate groups. The non-endotoxic lipid A variants are usually under-acylated, and/or possess longer (16 to 18 carbon atoms) lipid chains and lack at least one of the phosphate groups. The TLR4/MD-2 receptor complex responds to very low concentrations (picomolar magnitudes) of LPS *via* recognition and binding of distinct structural motifs of lipid A through majorly hydrophobic, but also ionic interactions (44). In this study, ESRD patients and HV differ in their LPS composition according to the length of the lipid A carbon chains: ESRD patients have more LPS with 12-14-carbons, and less LPS with 18-carbons as compared with healthy individuals. We tested the effect of HV and patient sera on cytokine secretion in an *in vitro* culture model of monocytes. We observed that the predominance of LPS with 18-carbons was associated with a lower monocyte-induced inflammation, whereas the predominance of LPS with 12-14-carbons induced a higher secretion of pro-inflammatory cytokines. Moreover, using TLR4 pathway antagonists, we demonstrated that monocyte-induced cytokine production was LPS-specific. In advanced chronic kidney disease, uremia may impair the integrity of the intestinal tight junctions, leading to an increased intestinal permeability and therefore endotoxemia (45). The disruption of the normal symbiotic relationship and alteration of the gut microbiota can also contribute to systemic inflammation and uremic toxicity (2). The composition difference of circulating LPS could rather come from the difference in the gut microbiota composition between HV and ESRD patients. Indeed, decrease in diversity and modifications in phyla distribution have been associated with pathological settings (46–48). Use of antibiotics and uremic toxins are known to profoundly modify the diversity and equilibrium between phyla that compose gut microbiota (38). Thus, the difference in circulating LPS subtype proportions between HV and ESRD should be analyzed with regards to the reciprocal microbiota composition. Dysbiosis (38, 49) can explain the composition difference in LPS subtypes, and consequently LPS-induced inflammation in these patients, with the passage of a lower proportion of C18 LPS –a LPS subtype with a potential anti-inflammatory function– in healthy context.

Several studies have shown that LPS clearance rates can markedly influence the inflammatory and immune response *in vivo* (50–52). Calculation of the LPS activity to mass ratio is a relevant method to assess the intrinsic capacity of plasma to neutralize the LPS activity by lipoprotein-mediated transport to the liver. In this study, the ratio was lower in healthy individuals, suggesting that LPS subtypes were better neutralized. Dialysis patients are known to present a dyslipidemia with hypertriglyceridemia, elevated lipoprotein-a, low-HDL and reduced apolipoprotein C-I (53, 54). In this study, 57 patients were dialyzed (83.8%) and lower HDL concentration may account for a less effective LPS clearance in ESRD patients. Indeed, cholesterol can be excreted in the bile *via* the process of reverse transport of cholesterol from peripheral tissues to the liver through the HDL pathway. Moreover, HDL and PLTP are the leading components of the ‘reverse LPS transport’, an *in vivo* LPS detoxification pathway that enhances LPS elimination as the ultimate and irreversible detoxification process in the bile (55). The presence of pro-inflammatory circulating LPS could increase the PLTP activity in ESRD, in order to transport LPS to HDL, but low HDL concentration reduces the capacity of LPS neutralization in these patients as compared with healthy individuals, leading to a persistent inflammation state.

Our study has some limitations. The measure of circulating bacterial products was based on the detection of circulating bacterial products on LAL and quantitative LPS by HPLC measurements. Unfortunately we did not have enough material to measure circulating 16sDNA. Monocyte inflammatory response to ESRD and HV sera could only be evaluated on HV-derived monocytes, since we did not have fresh samples from ESRD patients. Moreover, we used sera as LPS source for the *in vitro* experiments without considering the possible influence of other serum factors and/or clinical characteristics of each ESRD patient and HV. A method of extraction and isolation of each LPS subtypes must be developed to overcome this lack of samples purity. Finally, we only evaluated the implication of Gram-negative bacteria in GBT through the LPS analysis; further investigation of gut microbiota composition in ESRD remains to be studied to complete the GBT exploration. Diabetic nephropathy was underrepresented among nephropathy etiologies because this study included during a one-year period all consecutive ESRD patients on the kidney transplant waiting list who were called to be transplanted in our kidney transplant center. Of note, diabetic ESRD patients accounted for 16/68 (24%) patients (**Table 1**). The etiology of nephropathy among diabetic ESRD patients were: diabetes in 5/16, chronic glomerulopathy in 2/16, polycystic kidney disease in 1/16, other nephropathy in 7/16 and undetermined in 1/16. In our country, according to the national registry, diabetes represented only 8% of the etiologies of nephropathies among ESRD patients on the transplant waiting list in 2018 (56) because of cardiovascular contraindications. Thus, our ESRD cohort was representative of the national frequency of the etiologies declared for this population, yet our results could not be fully extrapolated to the whole population of ESRD patients.

**Figure 7:**
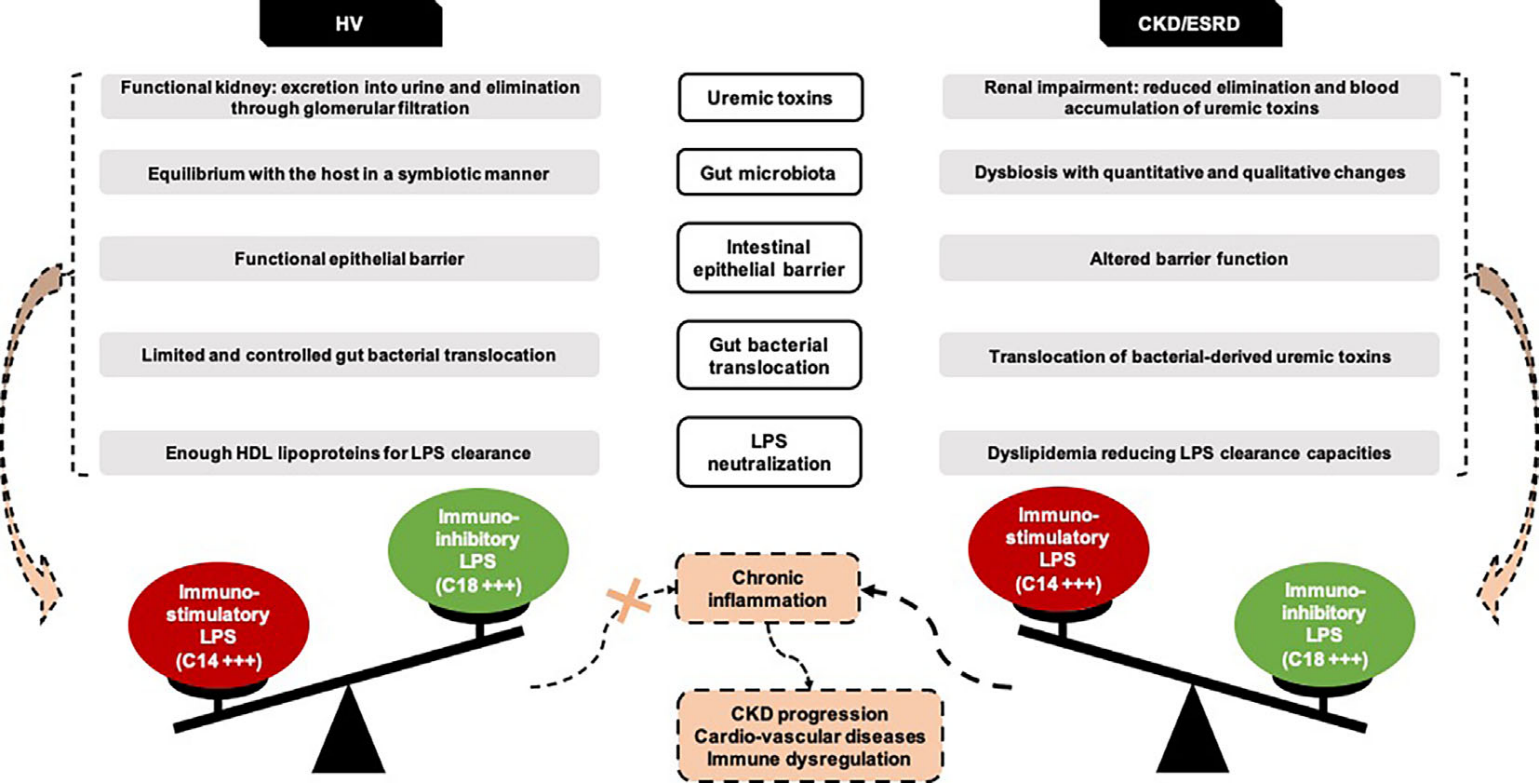
New insights on ESRD and HV gut bacterial translocation. Representation of the hypotheses emerging from the GABII cohort study. CKD, Chronic Kidney Disease; ESRD, End-Stage Renal Disease; HV, Healthy Volunteers.

Thereby, this study is the first to demonstrate that GBT is not a pathogenic state in ESRD. Indeed, septic inflammation observed in ESRD is not related to higher amount of GBT but to difference in circulating LPS subtypes and to a lesser capacity of LPS neutralization compared to healthy individuals (**Figure 7**). This suggests that interventions should focus on both modification of gut microbiota –which affects directly circulating LPS subtypes– and improvement in LPS neutralization.

## Data Availability Statement

The original contributions presented in the study are included in the article/**Supplementary Material**. Further inquiries can be directed to the corresponding author.

## Ethics Statement

The study was approved by the local Ethics Committee (CPP-EST-II, Besançon, France): favorable opinion ethic 14/438 of May 13th, 2014 and regulatory authorization n°140578B-11 of July 18th, 2014 (ANSM reference: 2014-A00424-43). The patients/participants provided their written informed consent to participate in this study. Written informed consent was obtained from the individual(s) for the publication of any potentially identifiable images or data included in this article.

## Author Contributions

HA-R, CC, PS, LL, EG, JB, and DD participated to the study design. HA-R, CC, JB, DD, DS-F, and PL participated in patients’ recruitment and data acquisition. HA-R, JB, DD, and PS participated in writing the article. J-PP, VD, HC, EC, and A-LR performed the LPS, HDL-cholesterol and cytokines analyses. HA-R and CC performed the sCD14 analyses. HA-R performed the *in vitro* experiments. CL performed the samples storage. All authors contributed to the article and approved the submitted version.

## Funding

This work was supported by grants from the Fondation Transplantation, the PHRC 2005 and 2011 (to DD), the Fondation de France (Appel d’offre “Maladies Cardiovasculaires” 2007 No. 2007 001859 [to PS]), the DHOS/INSERM/INCa (Appel d’offre Recherche Translationnelle 2008 [to DD and PS]), and the APICHU 2010 and 2014 (to JB), from the Agence Nationale de la Recherche (Labex LipSTIC, ANR-11-LABX-0021) and the Région de Franche-Comté (support to Labex LipSTIC [to PS] 2018 and 2019). JB received financial support from the Fondation Transplantation (No. ET-031211 and No. ET-050320). CC received PhD grant from the University of Franche-Comté. HA-R received PhD grant from the Région de Franche-Comté. This work is a part of the RIALTO (Research in Immunology of AtheroscLerosis after TransplantatiOn) program. This work is supported by the Fédération Hospitalo-Universitaire INCREASE (INtegrated Centre for Research in Inflammatory DisEASEs).

## Conflict of Interest

The authors declare that the research was conducted in the absence of any commercial or financial relationships that could be construed as a potential conflict of interest.
