# Supplementary material for: New Insights on End-Stage Renal Disease and Healthy Individual Gut Bacterial Translocation: Different Carbon Composition of Lipopolysaccharides and Different Impact on Monocyte Inflammatory Response
Source: Front Immunol. 2021 Jun 7;12:658404. doi: 10.3389/fimmu.2021.658404 (PMC8215383; doi:10.3389/fimmu.2021.658404)
Supplement: Supplementary file 1 [file DataSheet_1.docx]

# SUPPLEMENTARY FILES

**Supplementary figures**

**Figure S1 | Correlation between LPS subtype’s proportion with sCD14 in healthy volunteers (HV)**. Correlation between sCD14 serum levels and 3OHC12:0 **(A)**, 3OHC14:0 **(B)**, 3OHC16:0 **(C)** or 3OHC18:0 **(D)** LPS proportions in HV (n=20). Each cross represents a healthy individual. Linear regression was displayed on graphs. Spearman correlation between sCD14 and LPS proportions, **p*<0.05, ***p*<0.01, ****p*<0.001. LPS, lipopolysaccharide; sCD14, soluble cluster of differentiation 14.

**Figure S2 | TLR4-dependent inflammation pathway in end-stage renal disease (ESRD) patients and healthy volunteers (HV).** Human monocytes were stimulated with ESRD patient’s serum according to the highest proportion of each LPS subtypes (P/C12, P/C14, P/C16, P/C18, n=3 for each), with HV serum (n=5), or with decomplemented autologous serum and LPS from *E. coli* (LPS-EK, 10ng/mL, n=3) for the positive control. Moreover, cells and sera were cultured with an antagonist of TLR4 (LPS from *R. sphaeroides*, LPS-RS, 100 ng/mL) or an antibiotic blocking LPS activity (Polymyxin B, PMB, 100 µg/mL). After 12 hours of stimulation, supernatants were collected and the secretion of cytokines TNF-α **(A)**, IL-1β **(B)**, IL-6 **(C)** and IL-10 **(D)** were measured by enzyme-linked immunosorbent assay (ELISA). Bars and errors bars are expressed as median with interquartile range. Mann-Whitney U test of single stimulation (LPS-EK or sera) and co-stimulation with PMB or LPS-RS values, **p*<0.05, ***p*<0.01, ****p*<0.001, *****p*<0.0001, ns: not significant. IL, interleukin; LPS, lipopolysaccharide; TLR4, Toll-like receptor-4; TNF, tumor necrosis factor.


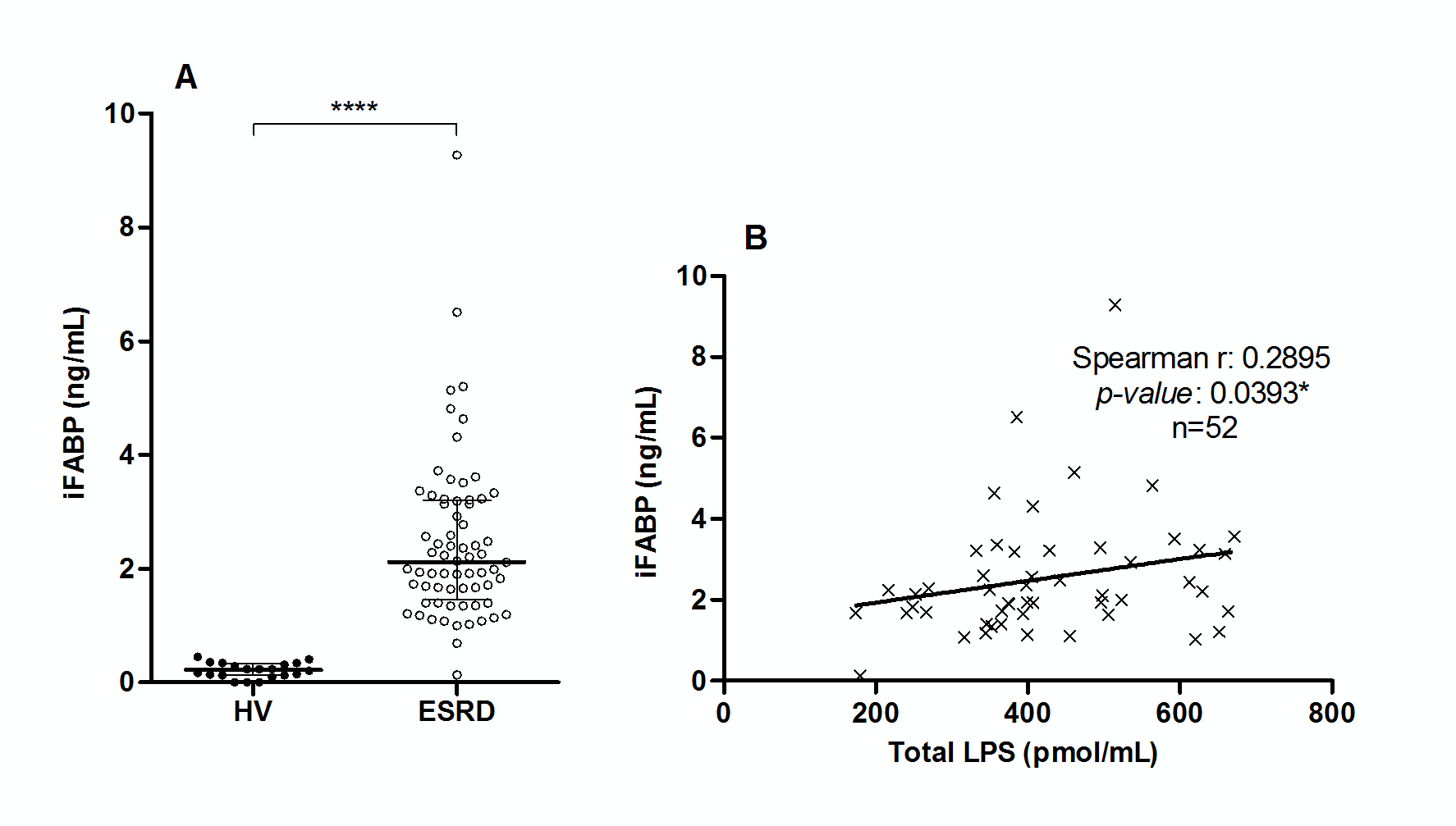


**Figure S3 | Disruption of the intestinal epithelial barrier in end-stage renal disease (ESRD) patients *versus* healthy volunteers (HV). (A)** Comparison of plasma iFABP, a biomarker of intestinal barrier dysfunction, between ESRD patients (n=68) and healthy volunteers (n=20). iFABP was determined in serum by enzyme-linked immunosorbent assay (ELISA). Bars and errors bars are expressed as median with interquartile range. Mann-Whitney U test of HV *versus* ESRD patient’s values, **p*<0.05, ***p*<0.01, ****p*<0.001, *****p*<0.0001, ns: not significant. **(B)** Correlation between plasma iFABP and total circulating LPS, a biomarker of GBT, in ESRD patients excluding diabetic patients (n=52). LPS quantification in serum was performed by HPLC/MS/MS method. Each cross represents a patient. Linear regression was displayed on graph. Spearman correlation between circulating iFABP and total LPS concentrations, **p*<0.05, ***p*<0.01, ****p*<0.001. GBT, gut bacterial translocation; HPLC/MS/MS, high performance liquid chromatography (HPLC) coupled with mass spectrometry (MS/MS); iFABP, intestinal fatty-acid binding protein; LPS, lipopolysaccharide.

**Supplementary tables**

**Table S1 | Difference in the concentrations and percentages of LPS subtypes according to the length of the carbon chain composing the lipid A in ESRD patients and healthy volunteers.**

| **LPS Subtypes** | **ESRD patients** | **Healthy volunteers** | ***p*** |
| --- | --- | --- | --- |
| C12 LPS concentrations (pmol/ml) | 51.4 [36.4–69.2] | 79.6 [62.0–84.8] | 0.0007 |
| C12 LPS percentage (%) | 12.6 [9.4–15.4] | 8.9 [7.2–10.5] | 0.0019 |
| C14 LPS concentrations (pmol/ml) | 50.7 [36.7–71.2] | 63.3 [47.6–70.2] | 0.1368 |
| C14 LPS percentage (%) | 11.9 [9.7–16.1] | 7.3 [6.1–9.1] | <0.0001 |
| C16 LPS concentrations (pmol/ml) | 228.6 [192.8–269.5] | 392.2 [322.4–423.6] | <0.0001 |
| C16 LPS percentage (%) | 53.9 [49.9–57.7] | 46.5 [43.8–48.2] | <0.0001 |
| C18 LPS concentrations (pmol/ml) | 85.2 [64.2–117.3] | 304.5 [258.0–342.8] | <0.0001 |
| C18 LPS percentage (%) | 19.9 [17.2–23.4] | 37.1 [35.4–38.2] | <0.0001 |

ESRD, end-stage renal disease patient (n=68); LPS, lipopolysaccharide; healthy volunteers (n=20); data are expressed as median [interquartile].

**Table S2 | Circulating sCD14 and pro-inflammatory cytokines in ESRD patients and healthy volunteers.**

| **Cytokines** | **ESRD patients** | **Healthy volunteers** | ***p*** |
| --- | --- | --- | --- |
| sCD14 (µg/mL) | 3.390 [2.86–3.95] | 2.251 [2.00–2.64] | <0.0001 |
| TNF-α (pg/ml) | 1.684 [0.00–5.71] | 0.00 [0.00–0.52] | 0.0040 |
| IL-1β (pg/ml) | 0.00 [0.00–8.63] | 0.00 [0.00–0.00] | 0.0028 |
| IL-6 (pg/ml) | 1.773 [0.43–5.71] | 0.00 [0.00–0.00] | <0.0001 |

ESRD, end-stage renal disease patient (n=68); healthy volunteers (n=20); IL, interleukin; sCD14, soluble form of cluster of differentiation 14; TNF, tumor necrosis factor; data are expressed as median [interquartile].

**Table S3 | Characteristics of end-stage renal disease (ESRD) patients from GABII cohort according to the clusters defined according to C18/(C12+C14+16) LPS ratio.**

| **GABII cohort** | **Cluster 1**  ***center value = 22%*** | **Cluster 2**  ***center value = 42%*** | ***p*** |
| --- | --- | --- | --- |
| **N** | 51 | 17 | _ |
| **Age (years)** | 52±15 | 49±14 | 0.543 |
| **Male gender (%)** | 32 (63) | 11 (64.5) | 0.986 |
| **BMI (kg/m²)** | 26±5 | 27±6 | 0.695 |
| **Dialysis duration (months)** | 30±25 | 40±26 | 0.224 |
| **Diabetes n. (%)** | 11 (22) | 5 (29) | 0.778 |
| **Hypertension n. (%)** | 50 (98) | 17 (100) | 0.572 |
| **Dyslipidemia n. (%)** | 27 (53) | 9 (50) | 0.840 |
| **Current smokers n. (%)** | 10 (20) | 5 (31) | 0.358 |
| **Medication with statins n. (%)** | 21 (42) | 7 (38) | 0.755 |
| **Medication with platelet antiaggregant n. (%)** | 9 (18) | 1 (6) | 0.175 |
| **Medication with phosphate-binding agent n. (%)** | 32 (63) | 10 (59) | 0.015 |
| **Anti-HLA immunization n. (%)** | 16 (31) | 1 (6) | 0.008 |
| **CKD-EPI Estimated GFR (ml/min/1.73m2)** | 12±4 | 11.5±6.0 | 0.929 |
| **sCD14 (µg/ml)** | 3.54 [3.02-3.96] | 2.86 [1.93-3.50] | 0.002 |
| **TNF-𝛼 (pg/ml)** | 1.80 [0-8.0] | 0 [0-2.5] | 0.028 |
| **IL-8 (pg/ml)** | 119 [3.40-664] | 26.56 [9.30-264] | 0.027 |
| **IL-7 (pg/ml)** | 2.74 [1.20-5.56] | 1.64 [1.33-2.02] | 0.002 |
| **Total LPS (pmol/ml)** | 399 [341-485] | 538 [410-669] | 0.0001 |
| **C12 LPS (%)** | 13 [9.45-16.24] | 10 [9.26-12.84] | 0.037 |
| **C14 LPS (%)** | 12 [10.35-16.62] | 10.5 [9.04-10.71] | 0.052 |
| **C16 LPS (%)** | 55 [51.70-58.10] | 49 [44.96-51.77] | 0.0001 |
| **C18 LPS (%)** | 19 [16.68-20.97] | 28 [24.74-32.93] | 0.0001 |

Two clusters among ESRD patients were defined according to C18/(C12+C14+C16) LPS ratio. Cluster 1 (n=51) was defined with a center value of 22% and cluster 2 (n=17) with a center value of 42% (*p*<0.001). Results are presented as mean ± SD. BMI: body mass index; CKD-EPI: chronic kidney disease - epidemiology collaboration; GABII: *Globulines Anti-lymphocytaires polyclonales et Barrière Immunitaire Intestinale*; GFR: glomerular filtration rate; HLA: human leukocyte antigen.

**Table S4 | Selection of ESRD sera according to the highest proportion of each LPS subtypes.**

| **Patient**  **number** | **Patient**  **identification** | **LPS subtypes (% of total LPS)** | | | |
| --- | --- | --- | --- | --- | --- |
|  |  | **3OHC12:0** | **3OHC14:0** | **3OHC16:0** | **3OHC18:0** |
| **36** | **P/C12-1** | **22.41** | 9.60 | 49.97 | 18.02 |
| **52** | **P/C12-2** | **26.00** | 28.40 | 44.76 | 0.84 |
| **44** | **P/C12-3** | **20.44** | 9.36 | 51.04 | 19.17 |
| **52** | **P/C14-1** | 26.00 | **28.40** | 44.76 | 0.84 |
| **1** | **P/C14-2** | 12.53 | **27.01** | 53.37 | 7.10 |
| **4** | **P/C14-3** | 20.15 | **23.14** | 49.14 | 7.57 |
| **54** | **P/C16-1** | 5.85 | 19.19 | **67.76** | 7.20 |
| **56** | **P/C16-2** | 2.71 | 16.87 | **70.74** | 9.68 |
| **42** | **P/C16-3** | 16.01 | 9.90 | **66.78** | 7.30 |
| **64** | **P/C18-1** | 17.09 | 8.17 | 39.68 | **35.06** |
| **65** | **P/C18-2** | 9.84 | 6.49 | 46.04 | **37.63** |
| **66** | **P/C18-3** | 6.06 | 12.53 | 45.12 | **36.29** |

ESRD, end-stage renal disease patient (n=68); LPS, lipopolysaccharide; P/C12, ESRD sera with the highest 3OHC12:0 LPS (n=3); P/C14, ESRD sera with the highest 3OHC14:0 LPS (n=3); P/C16, ESRD sera with the highest 3OHC16:0 LPS (n=3); P/C18, ESRD sera with the highest 3OHC18:0 LPS (n=3).

**Table S5 | Clinical characteristics of selected ESRD sera according to the highest proportion of each LPS subtypes.**

| **GABII cohort** | **Selected sera of ESRD patients** | | | | | | | | | | | |
| --- | --- | --- | --- | --- | --- | --- | --- | --- | --- | --- | --- | --- |
|  | **P/C12** | | | **P/C14** | | | **P/C16** | | | **P/C18** | | |
|  | **1** | **2** | **3** | **1** | **2** | **3** | **1** | **2** | **3** | **1** | **2** | **3** |
| **Age (years)** | 57 | 67 | 46 | 67 | 60 | 47 | 48 | 69 | 24 | 56 | 54 | 71 |
| **Gender (M or F)** | M | M | M | M | M | F | M | M | M | M | F | M |
| **BMI (kg/m²)** | 25.3 | 26.9 | 23.9 | 26.9 | 29.1 | 26.0 | 25.3 | 22.5 | 24.5 | 25.7 | 34.3 | 24.7 |
| **Dialysis** |  |  |  |  |  |  |  |  |  |  |  |  |
| **Dialysis method (HD or PD)** | HD | HD | HD | HD | HD | HD | HD | PD | PD | HD | PD |  |
| **Dialysis duration (months)** | 64 | 48 | 72 | 48 | 24 | 26 | 21 | 65 | 17 | 20 | 1 |  |
| **Previous kidney transplantation** |  |  |  |  |  |  |  |  |  |  |  |  |
| **Diabetes** |  |  |  |  |  |  |  |  |  |  |  |  |
| **Hypertension** |  |  |  |  |  | *MD* |  |  |  |  |  |  |
| **Dyslipidemia** |  |  |  |  |  |  |  |  |  |  |  |  |
| **Current smoker** |  |  |  |  |  |  |  |  |  |  |  |  |
| **Medication with statins** |  |  |  |  | *MD* |  |  |  |  |  |  |  |
| **Medication with platelet antiaggregant** |  |  |  |  |  |  |  |  |  |  |  |  |
| **Medication with phosphate-binding agent** |  |  |  |  |  |  |  |  |  |  |  |  |
| **Anti-HLA immunization** |  |  |  |  |  |  |  |  |  |  |  |  |
| **Etiology of kidney disease** |  |  |  |  |  |  |  |  |  |  |  |  |
| *Chronic glomerulopathy* |  |  |  |  |  |  |  |  |  |  |  |  |
| *Nephroangiosclerosis* |  |  |  |  |  |  |  |  |  |  |  |  |
| *Polykystic disease* |  |  |  |  |  |  |  |  |  |  |  |  |
| *Diabetes* |  |  |  |  |  |  |  |  |  |  |  |  |
| *Other hereditary nephropathy* |  |  |  |  |  |  |  |  |  |  |  |  |
| *Other nephropathy* |  |  |  |  |  |  |  |  |  |  |  |  |
| *Unknown* |  |  |  |  |  |  |  |  |  |  |  |  |
| **CKD-EPI Estimated GFR (ml/min/1.73m2)** | 5 | 11 | 4 | 11 | 5 | 6 | 11 | 4 | 3 | 6 | 6 | 16 |

The gray boxes correspond to the presence of the clinical characteristics for each selected ESRD patient. BMI: body mass index; CKD-EPI: chronic kidney disease - epidemiology collaboration; ESRD, end-stage renal disease patient; F, female; GABII: *Globulines Anti-lymphocytaires polyclonales et Barrière Immunitaire Intestinale*; GFR: glomerular filtration rate; HD, hemodialysis; HLA: human leukocyte antigen*;* LPS, lipopolysaccharide; M, male; MD, missing data; P/C12, ESRD sera with the highest 3OHC12:0 LPS (n=3); P/C14, ESRD sera with the highest 3OHC14:0 LPS (n=3); P/C16, ESRD sera with the highest 3OHC16:0 LPS (n=3); P/C18, ESRD sera with the highest 3OHC18:0 LPS (n=3); PD, peritoneal dialysis.

**Table S6 | Selection of HV sera according to the highest proportion of 3OHC18:0 LPS (n=5).**

| **HV**  **number** | **HV**  **identification** | **LPS subtypes (% of total LPS)** | | | |
| --- | --- | --- | --- | --- | --- |
|  |  | **3OHC12:0** | **3OHC14:0** | **3OHC16:0** | **3OHC18:0** |
| **4** | **HV-1** | 7.60 | 6.09 | 42.74 | **43.58** |
| **15** | **HV-2** | 8.15 | 6.09 | 43.41 | **42.35** |
| **19** | **HV-3** | 10.83 | 5.31 | 43.64 | **40.22** |
| **12** | **HV-4** | 6.97 | 7.17 | 47.38 | **38.49** |
| **10** | **HV-5** | 10.26 | 9.54 | 41.89 | **38.30** |

HV, healthy volunteers (n=20); LPS, lipopolysaccharide.
